# Toward Improving Interventions Against Toxoplasmosis by Identifying Routes of Transmission Using Sporozoite-specific Serological Tools

**DOI:** 10.1093/cid/ciaa428

**Published:** 2020-04-13

**Authors:** Gregory Milne, Joanne P Webster, Martin Walker

**Affiliations:** 1 Department of Pathobiology and Population Sciences, Royal Veterinary College, University of London, Hertfordshire, United Kingdom; 2 London Centre for Neglected Tropical Disease Research, Imperial College London Faculty of Medicine, St Mary’s Hospital Campus, London, United Kingdom

**Keywords:** *Toxoplasma gondii*, oocysts, TgERP, IgG, mathematical model

## Abstract

**Background:**

Horizontal transmission of *Toxoplasma gondii* occurs primarily via ingestion of environmental oocysts or consumption of undercooked/raw meat containing cyst-stage bradyzoites. The relative importance of these 2 transmission routes remains unclear. Oocyst infection can be distinguished from bradyzoite infection by identification of immunoglobulin G (IgG) antibodies against *T. gondii* embryogenesis-related protein (TgERP). These antibodies are, however, thought to persist for only 6–8 months in human sera, limiting the use of TgERP serology to only those patients recently exposed to *T. gondii*. Yet recent serological survey data indicate a more sustained persistence of anti-TgERP antibodies. Elucidating the duration of anti-TgERP IgG will help to determine whether TgERP serology has epidemiological utility for quantifying the relative importance of different routes of *T. gondii* transmission.

**Methods:**

We developed a serocatalytic mathematical model to capture the change in seroprevalence of non-stage-specific IgG and anti-TgERP IgG antibodies with human age. The model was fitted to published datasets collected in an endemic region of Brazil to estimate the duration of anti-TgERP IgG antibodies, accounting for variable age–force of infection profiles and uncertainty in the diagnostic performance of TgERP serology.

**Results:**

We found that anti-TgERP IgG persists for substantially longer than previously recognized, with estimates ranging from 8.3 to 41.1 years. The Brazilian datasets were consistent with oocysts being the predominant transmission route in these settings.

**Conclusions:**

The longer than previously recognized duration of anti-TgERP antibodies indicates that anti-TgERP serology could be a useful tool for delineating *T. gondii* transmission routes in human populations. TgERP serology may therefore be an important epidemiological tool for informing the design of tailored, setting-specific public health information campaigns and interventions.

## Teaser

Through analyses of publicly available human serological data, we identified a previously unrecognized persistence of *Toxoplasma gondii* sporozoite–specific antibodies. We discuss how sporozoite-specific serology can elucidate the transmission dynamics of *T. gondii* and help quantify the importance of transmission routes to inform public health initiatives.

Approximately one-third of the world’s human population is seropositive for the apicomplexan protozoan parasite *Toxoplasma gondii*. Members of the cat family (Felidae) are the only known definitive hosts of *T. gondii*, yet the parasite can infect all warm-blooded animals as secondary or intermediate hosts [1]. Toxoplasmosis can have a profound impact on human health, not only in terms of congenital disease in infants, severe pathologies in immunocompromised individuals (eg, organ transplant recipients and people with AIDS) [2], and acute, symptomatic infections in adults during outbreaks [3–5], but also through its association with a large burden of behavioral and neurological disorders, including schizophrenia, in immunocompetent individuals [6–9]. *Toxoplasma gondii* is also of major economic importance for the livestock industry, being responsible for approximately 23% of ovine abortions in Europe and the United States [10].

There are 3 predominant routes of *T. gondii* transmission: vertical transmission from mother to offspring, and 2 forms of horizontal transmission. Horizontal transmission involves either consumption of bradyzoite tissue cysts from infected secondary/intermediate hosts, or ingestion of oocysts shed in the feces of infected felids [11]. Understanding the relative significance of each route in different epidemiological settings has important implications. First, public health information campaigns and interventions aimed at limiting the risk of human infection will be most effective if they are closely aligned with local epidemiology. Second, whether an infection is of oocyst or bradyzoite origin may relate to disease severity and clinical outcome, and may therefore be important for prognosis and clinical management [3, 12].

Currently, the relative epidemiological predominance of oocyst vs bradyzoite transmission remains elusive. It has been proposed that due to a short duration of oocyst shedding and the time required for sporulation, this route is a minor contributor to human infection [13]. However, it is likely that the predominant horizontal infection route varies globally according to context-specific eating and hygiene practices [14]. For example, in Poland, where approximately 80% of livestock have been reported as *T. gondii* seropositive, bradyzoites from meat have been suggested to comprise a significant source of infection [15]. By contrast, in parts of Brazil, where many people live in poor socioeconomic conditions (and in a warm, humid climate favorable to long-term oocyst survival [16]), *T. gondii* infection has been largely attributed to oocysts [17].

The discovery that antibodies raised against an 11-kDa sporozoite-specific protein, *T. gondii* embryogenesis-related protein (TgERP), distinguish oocyst from bradyzoite infection has made investigations into the route of *T. gondii* transmission possible [18]. However, interpretation of results from this serological assay—and its potential use as an epidemiological tool to understand horizontal transmission—is hindered by the presumed short duration (6–8 months) of anti-TgERP immunoglobulin G (IgG) antibodies. However, recent serosurvey data [19, 20] showing a relatively high seroprevalence of anti-TgERP IgG, compared to non-stage-specific IgG, casts doubt on the transience of anti-TgERP IgG [21–23]. Mathematical modeling, by capturing the processes driving infection dynamics in populations, can help resolve this paradox and hence, in this context, can be used as a tool to estimate the duration of anti-TgERP IgG antibodies from seroprevalence data.

Here, we present a serocatalytic mathematical model that we fit to seroprevalence data from an endemic region of Brazil. We estimate the duration of anti-TgERP IgG antibodies and age–force of infection profiles and provide comprehensive sensitivity analyses to confirm the robustness of our findings to key assumptions. We discuss how the expanded use of TgERP serology in epidemiological studies will improve our understanding of the transmission dynamics of *T. gondii* and we highlight its potential as an epidemiological tool to inform the design of tailored, setting-specific public health initiatives.

## METHODS

### Epidemiological Setting and Serological Data

Age-stratified data on serum anti–*T. gondii* non-stage-specific IgG and anti-TgERP IgG were identified by searching Google Scholar, Web of Science, and PubMed using the terms “*Toxoplasma gondii* OR *T. gondii* OR *toxoplasmosis*” and “seroprev*,” along with either “oocyst*” or “sporozoite*.” Three studies were initially identified [19, 20, 24], 1 of which was excluded from the analysis due to a limited sample size (N = 10 for anti-TgERP IgG) [24]). The remaining 2 datasets were both collected in Campos dos Goytacazes, a municipality in the north of Rio de Janeiro State, Brazil, where *T. gondii* infection is highly endemic and primarily associated with the oocyst route of infection [17]. One dataset was generated using a stratified sampling approach to collect blood samples from individuals (N = 128) living in areas at different risk of groundwater contamination (hereafter referred to as “population data”) [19]. The other dataset was generated using a geographically representative sample of individuals associated with 10 regional public schools (N = 380), including students, students’ parents, and staff (hereafter referred to as “school data”) [20].

Non-stage-specific serum IgG was measured using a standard enzyme-linked immunosorbent assay (ELISA) for the school data and using a modified agglutination test (MAT) for the population data. For the population data [19], serum anti-TgERP IgG was measured using a standard deviation (SD) cutoff ELISA (where a sample is considered positive if its optical density is 3 SDs above the mean of seronegative controls) and, for the school data [20], using both an SD cutoff ELISA and a receiver operating characteristic (ROC) curve cutoff ELISA (where known-positive and -negative samples are analyzed and a suitable cutoff is chosen to optimize diagnostic performance). Seroprevalence estimates with 95% confidence intervals [25] are shown in Table 1.

**Table 1.** Seroprevalence of *Toxoplasma gondii* Measured by 2 Serological Diagnostics in Campos dos Goytacazes, Rio de Janeiro State, Brazil

Age Group, y	Non-Stage-Specific IgG Positive, % (95% CI)	Anti-TgERP IgG Positive Using ROC Curve Cutoff, % (95% CI)	Anti-TgERP IgG Positive Using SD Cutoff, % (95% CI)	Reference
8–19 (n = 23)	47.8 (29.2–67.0)	…	39.1 (22.1–59.3)	[19]
20–29 (n = 27)	66.7 (47.7–81.5)	…	55.6 (37.3–72.4)	
30–39 (n = 21)	90.5 (69.9–98.6)	…	52.4 (32.4–71.7)	
40–49 (n = 15)	73.3 (47.6–89.5)	…	46.7 (24.8–69.9)	
50–59 (n = 22)	100.0 (82.5–100.0)	…	54.5 (34.6–73.1)	
0–7 (n = 116)	11.2 (6.5–18.4)	31.0 (23.3–40.0)	7.8 (4.0–14.3)	[20]
8–14 (n = 156)	37.8 (30.6–45.6)	48.1 (40.4–55.9)	12.8 (8.4–19.0)	
15–21 (n = 48)	68.7 (54.6–80.1)	70.8 (56.7–81.8)	27.1 (16.5–41.1)	
22–28 (n = 60)	75.0 (62.7–84.3)	75.0 (62.7–84.3)	51.7 (39.3–63.8)	

IgG serological data were extracted from the literature from 2 studies in northern Rio de Janeiro State, Brazil. One study collected blood samples by stratified sampling of individuals [19] and the other by sampling individuals associated with 10 public schools [20]. The 95% binomial CIs were calculated using the Agresti-Coull method [25].

Abbreviations: CI, confidence interval; IgG, immunoglobulin G; ROC, receiver operating characteristic; SD, standard deviation; TgERP, *Toxoplasma gondii* embryogenesis-related protein.

### Mathematical Model

**Figure 1.**
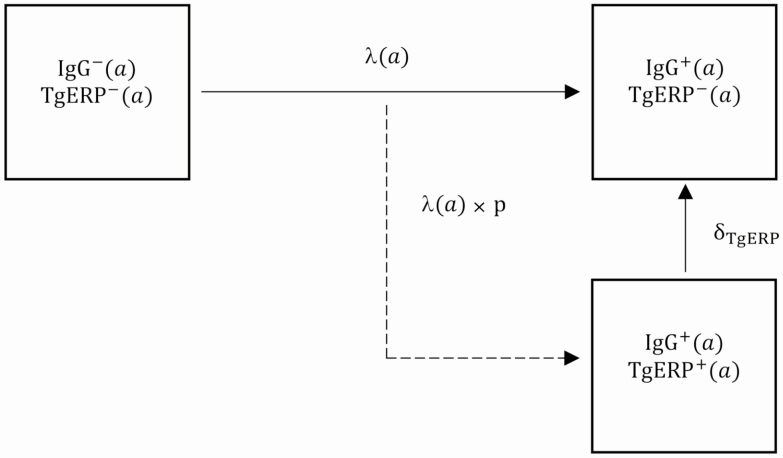
Serocatalytic model describing the age-seroprevalence dynamics of immunoglobulin G (IgG) and *Toxoplasma gondii* embryogenesis-related protein (TgERP) IgG antibodies. The compartments represent serostatus (seronegative or seropositive) for each of the 2 antibodies, anti–*T. gondii* non-stage-specific IgG and anti-TgERP IgG. The dynamics of the system are described by ordinary differential equations, based on transition rates (depicted by arrows) among states. Individuals beginning as seronegative for non-stage-specific IgG (IgG^−^) seroconvert to non-stage-specific IgG-positive (IgG^+^), at a rate determined by the age-specific force of infection λ(α). Individuals seroconvert from TgERP^–^ to TgERP^+^ at a rate defined by the product of λ(α) and the proportion of oocyst-derived infections, λ(α) × p. The rate of seroreversion of anti-TgERP is defined by δTgERP, where 1/δTgERP defines the duration of anti-TgERP antibodies. Non-stage-specific IgG antibodies are presumed to be lifelong and thus have no associated seroreversion parameter.

A serocatalytic model [26, 27] was built in R [28] based on a system of ordinary differential equations to estimate the duration of anti-TgERP IgG and age–force of infection profiles by fitting to the age-stratified serosurvey data (Supplementary Methods). The model describes age-seroprevalence profiles of non-stage-specific IgG and anti-TgERP IgG based on rates of seroconversion following exposure (and continuous reexposure) to *T. gondii* oocysts or bradyzoites and subsequent antibody-specific rates of seroreversion (Figure 1). We assumed that all individuals were born seronegative for both antibodies, and that the rate of seroconversion for each antibody was driven by an age-dependent force of infection (the per capita rate at which susceptible individuals acquire infection per unit time) and a parameter defining the proportion of oocyst-derived (as opposed to bradyzoite-derived) infections.

The force of infection was assumed to take 1 of 4 competing functional forms. The most complex, an exponentially damped relationship [29], initially increases with age to a peak and subsequently declines. The simpler functions describe, respectively, a linear relationship with age (with and without a positive intercept at birth) and a constant (age-independent) force of infection (Supplementary Methods and Supplementary Table 1). We varied the proportion of oocyst-derived infections between 0.50 and 1.0 in accordance with local epidemiological knowledge suggesting that oocysts account for the majority of infections in Campos dos Goytacazes [17]. The rate of seroreversion was assumed to be zero for non-stage-specific IgG, a common assumption indicative of a lifelong antibody response [21–23] (but see [30, 31] for alternative arguments). We estimated the rate of seroreversion (1/duration) of anti-TgERP IgG.

### Diagnostic Performance Characteristics

The modeled seroprevalence of non-stage-specific IgG and anti-TgERP IgG was adjusted for the imperfect sensitivity and specificity of the respective diagnostics [32]. Sensitivity and specificity of the non-stage-specific IgG ELISA (school data) was set at 94.5% and 98.7%, respectively, in accordance with published estimates (Supplementary Table 2). We also used these values for the sensitivity and specificity of the MAT (population data) because the MAT diagnostic performance is very similar to that of the ELISA (Supplementary Table 3).

The sensitivity and specificity of the ROC curve cutoff TgERP ELISA were set to 91.5% and 75.4%, respectively, in accordance with published estimates (Supplementary Table 2) [20]. Since no published estimates were available for the SD cutoff TgERP ELISA diagnostic values, a sensitivity analysis was performed, setting a range of values such that sensitivity for the SD cutoff ELISA was lower than the ROC curve cutoff ELISA and the specificity higher (since there is a higher proportion of positive cases in the ROC vs SD data [20] (Supplementary Table 4). For further details, see the Supplementary Methods.

### Maximum Likelihood Estimation of Model Parameters

A maximum likelihood approach was used to estimate the rate of seroreversion of anti-TgERP IgG and parameters defining the age–force of infection profile using a quasi-Newton procedure [33], assuming a binomial distribution for the data. For each dataset, the model was fitted simultaneously to the age-stratified seroprevalence of non-stage-specific IgG and anti-TgERP IgG (for the school dataset, this meant simultaneously fitting to both types of anti-TgERP data and non-stage-specific IgG data). The model was fitted repeatedly to the data using different functional forms of the age–force of infection profile, different assumed values of the proportion of oocyst-derived infections, and different diagnostic performance characteristics of the SD cutoff anti-TgERP ELISA. The likelihood of each fitted model was recorded and compared (using likelihood ratio tests for models with different age–force of infection functions) to determine the best-fitting yet most parsimonious model.

## RESULTS

### Age–Force of Infection Profiles

Across all combinations of the sensitivity and specificity of SD cutoff anti-TgERP ELISA, we found that the best-fitting age–force of infection profile (by likelihood ratio test) for the population data was consistently a constant (age-independent) force of infection; whereas for the school data, the best-fitting model was a linearly increasing function of age (with and without a positive intercept, depending the assumed sensitivity and specificity) (Table 2). For details, see Supplementary Tables 5 and 6.

**Table 2.** Comparison of Force of Infection Model Fits for Both Datasets

Age–Force of Infection	Population Data^a^	School Data
Exponentially damped^b^	−19.31	−44.08
Linear (positive intercept)	−19.32	−**44.08**
Linear (zero intercept)	−25.44	−47.41
Constant	−**19.32**	−55.18

Model were fitted to the population data [19] and school data [20] using a maximum likelihood approach and assuming a proportion of oocyst infections of 1. Bold text signifies the best-fitting model based on comparison of model log likelihoods using a likelihood ratio test. Given a nonsignificant test at the 5% level, the simpler model was considered an adequate explanation of the data. Parameter 95% confidence intervals were calculated by likelihood profiling [33]. Complete results for the population and school data are given in Supplementary Tables 5 and 6, respectively.

^a^Since model fit (log likelihood) was invariant with different *Toxoplasma gondii* embryogenesis-related protein enzyme-linked immunosorbent assay sensitivity and specificity values, a nominal set was chosen (80% and 95%, respectively). The complete results are shown in Supplementary Table 5.

^b^Initially increasing with age to a peak and subsequently declining [29].

### Proportion of Oocyst-derived Infections

For the population data [19], increasing the proportion of oocyst infections from 0.5 to 1 led to a consistent but small tendency for the log likelihood to increase across all age–force of infection profiles. For the school data [20], there was no consistent trend in the log likelihood with an increasing proportion of oocyst infections. However, estimates of anti-TgERP IgG duration did not fall within a plausible range until approximately 80% of infections were assumed to be oocyst-derived (ie, for < 80% oocyst infections, the duration of anti-TgERP IgG was estimated as longer than an average human lifespan). We therefore considered that the proportion of oocyst infections was likely to be high (consistent with local epidemiological knowledge [17]) and fixed this parameter to 1 for final inference on the rate of seroreversion (duration) of anti-TgERP antibodies. For details, see Supplementary Tables 7 and 8.

### Duration of Anti-TgERP IgG Antibodies

**Figure 2.**
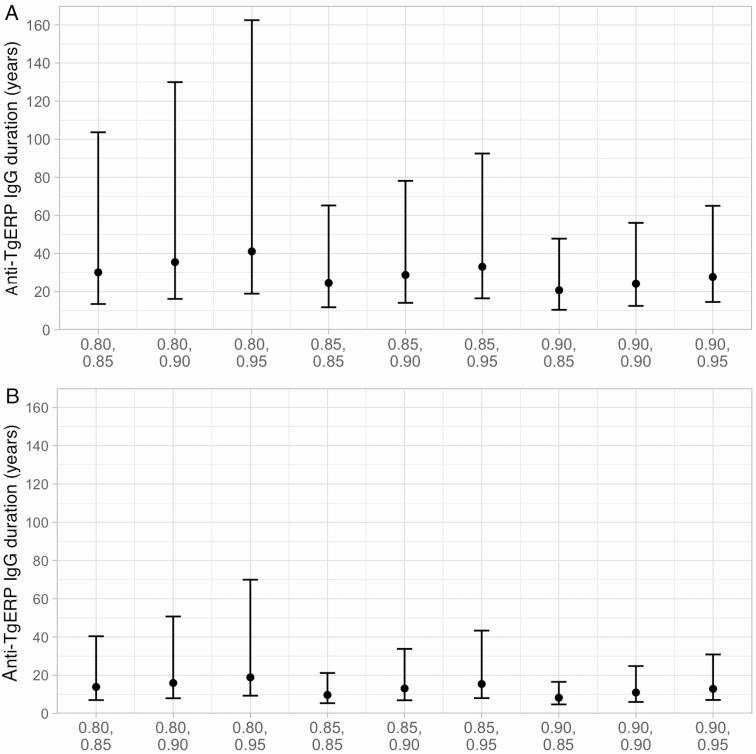
Sensitivity analysis for a range of different diagnostic values for the standard deviation cutoff *Toxoplasma gondii* embryogenesis-related protein (TgERP) enzyme-linked immunosorbent assay (x-axis format: sensitivity, specificity), assuming that all infections are oocyst-derived. Points and error bars show the maximum likelihood estimates for the duration of anti-TgERP immunoglobulin G (IgG) antibodies in years and 95% confidence intervals (estimated by likelihood profiling) [33]. *A*, Anti-TgERP IgG antibody duration estimates for the population data (N = 128) [13] for all combinations of diagnostic performance values (sensitivity, specificity) and the best-fit constant (age-independent) age–force of infection profile (Supplementary Table 5). *B*, Anti-TgERP IgG antibody duration estimates for the school data (N = 380) [15], for all combinations of diagnostic performance and the best-fitting linear age–force of infection profile (Supplementary Table 6).

**Figure 3.**
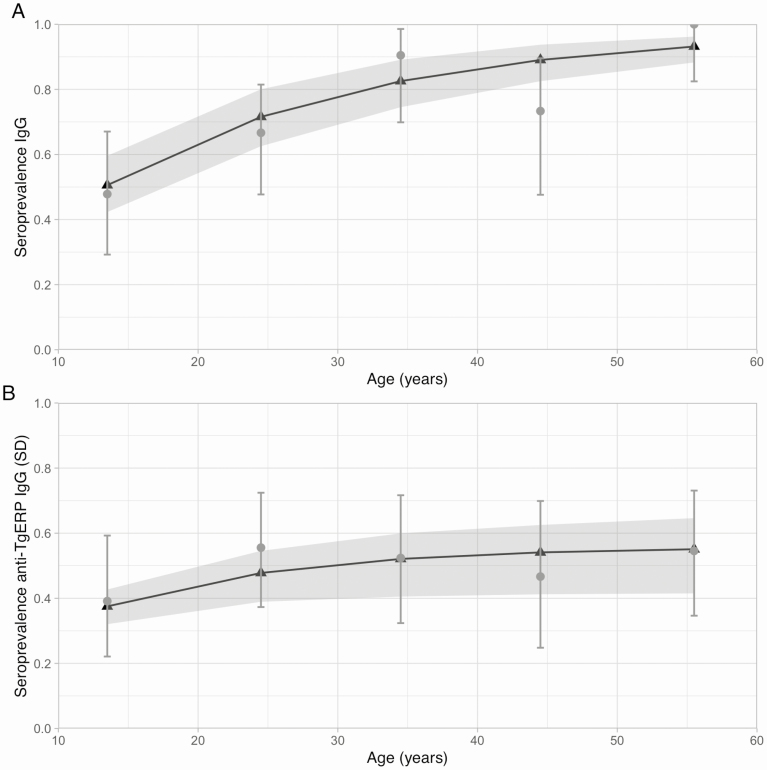
Fitted and observed age-seroprevalence of *Toxoplasma gondii* from a population sample of 128 individuals in Campos dos Goytacazes, Rio de Janeiro State, Brazil [19]. Seroprevalence data are shown in gray and model estimates in black, with respective 95% confidence intervals (CIs) given by error bars and shaded gray areas. Modeled seroprevalence estimates are adjusted by the sensitivity and specificity of the relevant diagnostic test and use the best-fitting constant (age-independent) force of infection relationship. CIs associated with the fitted model were constructed by sampling parameter values from their approximate sampling distribution (Supplementary Methods). Panels show the fitted vs observed seroprevalence of non-stage-specific immunoglobulin G (IgG), measured by modified agglutination test (*A*), and anti–*T. gondii* embryogenesis-related protein (TgERP) IgG measured by standard deviation (SD) cutoff enzyme-linked immunosorbent assay (*B*).

**Figure 4.**
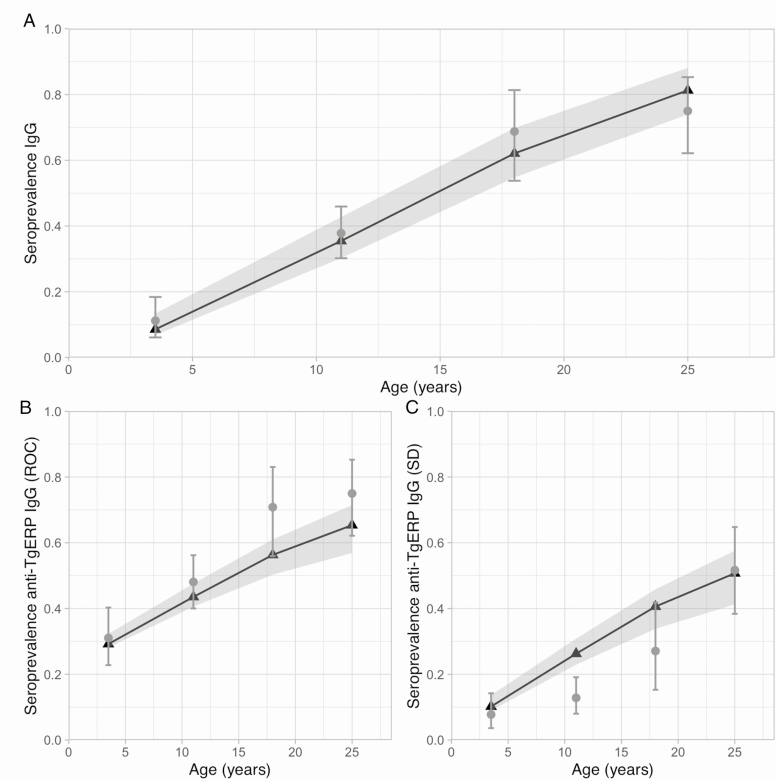
Fitted and observed age-seroprevalence of *Toxoplasma gondii* among 380 individuals associated with 10 public schools in Campos dos Goytacazes, Rio de Janeiro State, Brazil [20]. Seroprevalence data are shown in gray and model estimates in black, with respective 95% confidence intervals (CIs) given by error bars and shaded gray areas. Modeled seroprevalence estimates are adjusted by the sensitivity and specificity of the relevant diagnostic test and use the best-fitting (linear) force of infection relationship. CIs associated with the fitted model were constructed by sampling parameter values from their approximate sampling distribution (Supplementary Methods). Panels show the fitted vs observed seroprevalence of non-stage-specific immunoglobulin G (IgG), measured by enzyme-linked immunosorbent assay (ELISA) (*A*); anti–*T. gondii* embryogenesis-related protein (TgERP) IgG measured by receiver operating characteristic curve cutoff ELISA (*B*); and anti-TgERP IgG measured by standard deviation (SD) cutoff ELISA (*C*).

Maximum likelihood estimates of the duration (1/rate of seroreversion) of anti-TgERP IgG antibodies ranged between 20.7 and 41.1 years for the population data and between 8.3 and 18.9 years for the school data, depending on the assumed sensitivity and specificity of the SD cutoff TgERP ELISA, and using the best-fit age–force of infection profile. We found little variation in the likelihood of the data of different sensitivity and specificity values when fitting to the population data (Figure 2A and Supplementary Table 5), whereas for the school data the best-fitting model included an SD cutoff TgERP ELISA sensitivity and specificity of 80% and 95%, respectively, yielding a best point estimate for the duration of anti-TgERP IgG antibodies of 18.9 years (Figure 2B and Supplementary Table 6). The fitted non-stage-specific IgG and anti-TgERP IgG seroprevalence profiles compared to the observed school and population data are shown in Figures 3 and 4, respectively.

## DISCUSSION

Using a serocatalytic modeling approach incorporating the only substantial and publicly available TgERP serosurvey datasets [19, 20], we estimate that the duration of anti-TgERP IgG antibodies following exposure to *T. gondii* oocysts is between 8.3 and 41.1 years, substantially longer than the hitherto proposed 6–8 months [18]. If this finding can be confirmed in other epidemiological settings, the scope of TgERP serology may be greatly expanded from a predominantly clinical context, for patients with known recent exposure to *T. gondii*, to the epidemiological domain, to quantify the importance of the oocyst vs bradyzoite route of transmission in endemic communities. This could ultimately help to provide a stronger evidence base to inform public health initiatives aiming to control toxoplasmosis.

Hill and colleagues [18], who first demonstrated the use of TgERP serology to identify oocyst-derived infections, originally proposed the 6- to 8-month duration of the anti-TgERP IgG response. They estimated this using IgG avidity tests and TgERP serology on a small cohort of experimentally infected pigs (ie, with known time of exposure) in addition to historical human samples paired with information on probable timing of exposure.

However, since the IgG avidity index classification of chronic infection excludes primary infection in the last 3 months but has no upper limit for infection date [18], this estimate may have underestimated the duration of the anti-TgERP IgG response. Indeed, in line with a longer persistence of anti-TgERP IgG response, Vieira and colleagues [19] found that all anti-TgERP IgG–positive individuals were negative for immunoglobulin M (IgM), an observation strongly indicative of chronic infection. IgM titers usually persist for 6–9 months postinfection and in a small minority of cases have been shown to be detectable for 2 or more years [23]. These observations accord with our main finding, suggesting a previously unrecognized persistence of anti-TgERP IgG in oocyst-infected individuals.

Our results also indicate 2 distinct age–force of infection profiles, a constant (age-independent) [19] and linearly increasing [20] relationship with age, despite both analyzed datasets being collected from the same endemic region in Brazil. These differences have a number of possible explanations. First, since the 2 studies sampled individuals of very different age ranges, 8–59 years [19] and 0–28 years [20], the force of infection profiles could be consistent with a rapid increase in early childhood that later plateaus or decreases by adolescence and adulthood [34]. Second, while the population data were generated by stratified sampling of individuals from residential areas according to groundwater contamination risk [19], the school data were generated by sampling individuals associated with 10 regional public schools [20]. It is thus possible that these 2 study populations, despite coming from the same endemic setting, differ with respect to other unmeasured epidemiologically relevant variables.

Interestingly, for the model fitted to the school data [20], the best-fitting model contained a term for a nonzero force of infection at birth, which could be indicative of congenital infection. Indeed, in Brazil there is a very high rate of *T. gondii* vertical transmission, with 5–23 neonates congenitally infected per 10 000 births [35]. Given the force of infection estimated here—0.015 per person per year in newborns—this is equivalent to 150 neonates (30–270) congenitally infected per 10 000 births. This estimate is likely higher than that of a previous study for the whole of Brazil [35] due to the local epidemiological characteristics of this high-transmission-intensity setting, or because of systemic underreporting of congenital infection. Alternatively, since our model does not account for passive transmission of maternal IgG to neonates, this estimate could, in part, indicate the prevalence of maternally derived antibodies, rather than de novo antibody production following congenital infection [36]. Nonetheless, a nonzero force of infection at birth is unlikely due to setting-specific variation in diagnostic sensitivity or specificity, since the TgERP ROC curve cutoff diagnostic values were obtained directly from the school data [20]. Thus, our adjustment for imperfect diagnostic performance should provide a reasonably accurate measure of the true underlying force of infection. While the model is consistent with some level of congenital infection, without data on the seroprevalence in neonates, it is difficult to establish this with certainty.

We fully acknowledge that the mathematical model proposed here relies on a number of assumptions that could bias our estimates of the age–force of infection relationship and the duration of the anti-TgERP IgG response. First, it is assumed that the force of infection remains constant over time (note that this is distinct from age-associated changes, which are modeled explicitly [37]). A previous study incorporated time-related changes in the force of infection of *T. gondii* in Sweden, yielding lower incidence estimates of maternal toxoplasmosis than would be expected if the force of infection were temporally stable [29]. If the force of infection had been recently increasing very rapidly in the study setting, it is possible that the duration of anti-TgERP IgG could have been overestimated. However, in the absence of dramatic secular changes in environmental, socioeconomic, or cultural factors, there is little evidence to suggest this.

Second, the estimates presented here use data collected from a known high-transmission region of Brazil [17, 35], which may limit the generalizability of our findings. For example, it is possible that the duration of anti-TgERP IgG antibodies may be related to the cumulative lifetime exposure to *T. gondii* (TgERP) antigens rather than their production being continually “boosted” by repeated exposure, as is modeled here. This phenomenon is observed for the immune response to specific antigens of *Plasmodium falciparum* [38]. This hypothesis could be tested by adapting the current serocatalytic model to link antibody duration to cumulative exposure to *T. gondii* and the model fitted to seroprevalence data collected from settings with markedly different intensities of transmission. To date, there is only 1 study available in the literature conducted in a low-transmission region, in Scotland. However the study identified only 1 anti-TgERP IgG-positive case out of 10 individuals who had recently seroconverted [24] and hence had insufficient power for inclusion in our analyses. More abundant data on anti-TgERP seroprevalence from low-endemicity settings would thus be highly valuable for further understanding *T. gondii* antibody dynamics.

Third, South America has an unparalleled diversity of *T. gondii* genotypes, with an abundance of “atypical” genotypes rarely found elsewhere (those not conforming to the traditional type I–III designation) [35, 39, 40]. As TgERP plays a role in *T. gondii* embryogenesis, a key process in the sexual cycle, one may predict its expression to not differ significantly across parasite strains. In contrast, the duration of the antibody response may be expected to differ among strains and therefore show geographic heterogeneity. Hence we reiterate that a key next step is testing our findings using data from other geographic and epidemiological settings.

Serocatalytic modeling is used to analyze epidemiological data from a diverse array of pathogens [26] and, notably, for planning interventions against human malaria [41, 42], an apicomplexan cousin of *T. gondii*. Here we have applied this approach to estimate the duration of the anti-TgERP IgG response following exposure to *T. gondii* oocysts, finding that it is sustained for much longer than previously thought. If these findings can be validated in additional epidemiological settings, TgERP serology could potentially be used to quantify the relative importance of *T. gondii* transmission routes (oocyst vs bradyzoite) in endemic communities. Such setting-specific understanding would be valuable for informing public health information campaigns and designing tailored intervention strategies.

## Supplementary Data

Supplementary materials are available at *Clinical Infectious Diseases* online. Consisting of data provided by the authors to benefit the reader, the posted materials are not copyedited and are the sole responsibility of the authors, so questions or comments should be addressed to the corresponding author.

ciaa428_suppl_supplementary_Methods

## Notes

***Author contributions.*** G. M., J. P. W., and M. W. wrote the manuscript. All authors contributed to the design of the mathematical modeling and analysis.

***Financial support.*** This work was supported by the Biotechnology and Biological Sciences Research Council (London Interdisciplinary Doctoral Training Programme funding [number BB/M009513/1] to G. M., under the supervision of M. W. and J. P. W.) and the London International Development Centre (Interdisciplinary Seed Fund).

***Potential conflicts of interest.*** The authors: No reported conflicts of interest. All authors have submitted the ICMJE Form for Disclosure of Potential Conflicts of Interest.
