# Supplementary material for: Toward Improving Interventions Against Toxoplasmosis by Identifying Routes of Transmission Using Sporozoite-specific Serological Tools
Source: Clin Infect Dis. 2020 Apr 13;71(10):e686–93. doi: 10.1093/cid/ciaa428 (PMC7744992; doi:10.1093/cid/ciaa428)
Supplement: ciaa428_suppl_supplementary_Methods [file ciaa428_suppl_supplementary_methods.docx]

**Towards improving interventions against toxoplasmosis by identifying routes of transmission using sporozoite-specific serological tools**

**Supplementary Materials**

**METHODS**

**Mathematical model**

We define ${IgG}^{+}(a)$and ${IgG}^{-}\left( a \right)=1-{IgG}^{+}(a)$ as, respectively, the proportion of IgG-positive and IgG-negative individuals of age $a$. Similarly, ${TgERP}^{+}(a)$ and ${TgERP}^{-}\left( a \right)=1-{TgERP}^{+}(a)$ is the proportion of anti-*Toxoplasma gondii* embryogenesis related protein (TgERP) IgG-positive and IgG-negative individuals of age $a$. Assuming that the temporal dynamics of seroprevalence are at equilibrium (i.e. the force of infection is temporally stable), we can define the following system of coupled ordinary differential equations to describe changes in seroprevalence with age,

|  | $\frac{{IgG}^{+}(a)}{da}= (a) \cdot\left[ {1-IgG}^{+}(a) \right] - \delta_{IgG} \cdot{IgG}^{+}(a)$ | (1) |
| --- | --- | --- |
|  | $\frac{{TgERP}^{+}(a)}{da}=\left( a \right)\cdot p \cdot\left[ 1-{TgERP}^{+}(a) \right] - \delta_{TgERP} \cdot{TgERP}^{+}(a)$ | (2) |
|  |  |  |

Here, λ(*a*) denotes the age-dependent force of infection to *T. gondii* (either oocysts or bradyzoites), $\delta_{IgG}$ and $\delta_{TgERP}$ denote the rate of seroreversion of non-stage-specific IgG and anti-TgERP IgG respectively, and $p$ is the proportion of infections that result from exposure to oocysts. We modelled the age-force of infection profile, $(a$), using four nested functional forms,

|  | $(a)= {}_{0}+ {}_{1} \cdot(a \cdot e^{-ra})$ | (3) |
| --- | --- | --- |
|  | $(a)= {}_{0}+ {}_{1} \cdot a$ | (4) |
|  | $(a)= {}_{1} \cdot a$ | (5) |
|  | $(a)= {}_{0}$ | (6) |

where ${}_{0}$ is $(a=0)$ at birth, ${}_{1}$ is the initial rate of increase in $(a)$,and $r$is a shape term that determines the rate of decay of $(a)$ with age (Eq. (3) only). Hence, Eq. (3) defines an exponentially dampened relationship [1], Eq. (4) and Eq. (5) define a linear relationship, with and without a positive intercept, and Eq. (6) defines a constant force of infection with age (i.e. age-independent). A complete list of model parameters and definitions is given in Supplementary Table S1.

The “true” (unobserved) seroprevalence modelled by Eq. (1) and (2) was adjusted to account for the imperfect sensitivity and specificity of the diagnostics used to detect non-stage-specific IgG and anti-TgERP IgG (Supplementary Table S2), giving a modelled (“observed”) seroprevalence that could be fitted to the seroprevalence data [6]. We highlight that, while IgG antibodies for the population data [7] were detected using the modified agglutination test (MAT), we assumed that the sensitivity and specificity of the MAT was identical to the ELISA (used for the schools data [5]) on account of their similar performance characteristics in various (non-human) hosts (Supplementary Table S3).

The modelled (“observed”) non-stage-specific IgG seroprevalence as measured by conventional ELISA is denoted ${IgG}^{+^{'}}(a)$, and the anti-TgERP IgG as measured by receiver operating characteristic (ROC) curve cut-off ELISA or standard deviation (SD) cut-off ELSA is denoted by ${TgERP}_{ROC}^{+^{'}}(a)$ and ${TgERP}_{SD}^{+^{'}}(a)$ respectively. These quantities relate to the “true” (unobserved) seroprevalence by,

|  | ${IgG}^{+^{'}}(a)={IgG}^{+}(a) \cdot\left( {se}_{IgG}+ {sp}_{IgG}- 1 \right)+(1- {sp}_{IgG})$ | (7) |
| --- | --- | --- |
|  | ${TgERP}_{ROC}^{+^{'}}(a)={TgERP}^{+}(a) \cdot\left( {se}_{TgROC}+ {sp}_{TgROC}- 1 \right)+(1- {sp}_{TgROC})$ | (8) |
|  | ${TgERP}_{SD}^{+^{'}}(a)={TgERP}^{+}(a) \cdot\left( {se}_{TgSD}+ {sp}_{TgSD}- 1 \right)+(1- {sp}_{TgSD})$ | (9) |

where ${se}_{IgG}$, ${se}_{TgROC}$ and ${se}_{TgSD}$ correspond to the sensitivity of the conventional ELISA, anti-TgERP ROC curve and SD cut-off ELISAs, respectively, and ${sp}_{IgG}$, ${sp}_{TgROC}$ and ${sp}_{TgSD}$ to the corresponding specificities.

**Sensitivity analysis**

Since the sensitivity and specificity of the SD cut-off TgERP ELISA were unknown, we performed a sensitivity analysis to assess the influence of these parameters on the estimates of our main parameters of interest (rate of seroreversion of anti-TgERP IgG and parameters defining the force of infection). Three values were chosen for each sensitivity and specificity (on the premise that sensitivity for the SD ELISA should be lower than the TgERP ROC curve cut-off ELISA and the specificity higher), giving a total of nine possible pairs (Supplementary Table S4).

Maximum likelihood estimation was performed for each pair of sensitivity and specificity values for each of the four force of infection models. We used likelihood ratio tests (LRTs) to determine the best-fitting model for each pair of diagnostic performance values. For each comparison, we defined the deviance of the simpler and more complex models, respectively, as $D_{s}= -2\ln\mathcal{L}_{s}$ and $D_{g}= -2\ln\mathcal{L}_{g}$. The likelihood ratio is given by $D_{s}- D_{g}$, which follows a $\chi^{2}$(chi-squared) distribution, with degrees of freedom given by the difference in the number of parameters between the simpler and more complex model.

**Uncertainty**

To assess the uncertainty associated with the fitted model, we used a parametric bootstrap approach to simulate parameter values from a multivariate normal distribution, parameterised using the estimated variance-covariance matrix (the inverse of the hessian matrix) and coefficient point-estimates. We sampled 1,000 parameter values, calculating the age-specific (fitted) seroprevalence for each and constructing 95% confidence intervals by excluding values less than the 2.5 or greater than the 97.5 percentiles.

**Supplementary Table 1. Model parameters and definitions**

| Parameter | Description |
| --- | --- |
| $\delta_{IgG}$ | Rate of seroreversion of non-stage-specific anti-*Toxoplasma gondii* IgG |
| $\delta_{TgERP}$ | Rate of seroreversion of anti-TgERP IgG |
| $p$ | Proportion of oocyst-derived infections |
| ${}_{0}$ | Force of infection at birth |
| ${}_{1}$ | Initial rate of increase of the force of infection with age |
| $r$ | Shape term that allows force of infection to decay in older age groups (exponentially damped model) |

Abbreviations: IgG, immunoglobulin G; ELISA, enzyme-linked immunosorbent assay; TgERP, *Toxoplasma gondii* embryogenesis-related protein.

**Supplementary Table S2. ELISA sensitivity and specificity values for the detection of anti-*Toxoplasma gondii* IgG and anti-TgERP IgG in human sera**

|  | Sensitivity (%) | Specificity (%) | Reference |
| --- | --- | --- | --- |
| Anti-*T. gondii* IgG ELISA | 98.9 | 98.3 | [2] |
|  | 95.6 | 98.7 | [2] |
|  | 100 | 98.5 | [3] |
|  | 99.4 | 99.4 | [4] |
| Unweighted mean | 98.5 | 98.7 |  |
| Anti-TgERP IgG ELISA (ROC curve cut-off) | 92.3 | 76.7 | [5] |
|  | 91.5 | 78.4 |  |
|  | 90.9 | 73.3 |  |
|  | 91.1 | 73.3 |  |
| Unweighted mean | 91.5 | 75.4 |  |

Abbreviations: IgG, immunoglobulin G; ELISA, enzyme-linked immunosorbent assay; TgERP, *Toxoplasma gondii* embryogenesis-related protein; ROC, receiver operating characteristic.

**Supplementary Table S3. Sensitivity and specificity of modified agglutination test (MAT) compared to ELISA**

| MAT se (%) | MAT sp (%) | ELISA se (%) | ELISA sp (%) | Host (sample; n) | Reference |
| --- | --- | --- | --- | --- | --- |
| 92.6 | 95.5 | 90.5 | 97.8 | Sheep (sera; 203) | [8] |
| 76.0 | 68.0 | 85.0 | 56.0 | Chickens (sera; 135) | [9] |
| 85.7 | 94.6 | 88.6 | 98.0 | Pigs (sera, diaphragm fluid; 264) | [10] |
| 87.0 | 100.0 | 94.3 | 100.0 | Pigs (sera; 10) | [11] |
| 96.0 | 88.9 | - | - | Sheep (sera; 300) | [12] |
| 80.64 | - | 100.0 | - | Pigs (sera, heart and tongue tissue; 35) | [13] |
| 82.9 | 90.29 | 72.9 | 85.9 | Pigs (sera; 1000) | [14] |

Abbreviations: se, sensitivity; sp, specificity; IgG, immunoglobulin G; MAT, modified agglutination test; ELISA, enzyme-linked immunosorbent assay; TgERP, *Toxoplasma gondii* embryogenesis-related protein.

A comparison of the sensitivity and specificity of the MAT with the ELISA for detecting anti-*Toxoplasma gondii* IgG in various hosts. Data unavailable in humans.

**Supplementary Table S4.** **Standard deviation (SD) cut-off ELISA sensitivity and specificity values**

|  | Sensitivity (%) | Specificity (%) |
| --- | --- | --- |
| Minimum | 80 | 85 |
| Medium | 85 | 90 |
| Maximum | 90 | 95 |

Abbreviations: ELISA, enzyme-linked immunosorbent assay; SD, standard deviation.

Chosen sensitivity and specificity values for TgERP SD cut-off ELISA for the sensitivity analysis.

**Supplementary Table S5.** **Sensitivity analysis of a range of values for sensitivity and specificity of TgERP standard deviation (SD) cut-off ELISA (population data)**

|  | Sensitivity and specificity of TgERP SD cut-off ELISA | | | | | | | | |
| --- | --- | --- | --- | --- | --- | --- | --- | --- | --- |
|  | Set 1 | Set 2 | Set 3 | Set 4 | Set 5 | Set 6 | Set 7 | Set 8 | Set 9 |
| Model 1^a^ | -19.14 | -19.21 | -19.31 | -19.12 | -19.17 | -19.24 | -19.11 | -19.13 | -19.19 |
| Model 2^b^ | -19.14 | -19.21 | -19.32 | -19.12 | -19.17 | -19.25 | -19.12 | -19.15 | -19.21 |
| Model 3^c^ | -23.95 | -24.62 | -25.44 | -23.99 | -24.67 | -25.50 | -24.04 | -24.72 | -25.56 |
| Model 4^d^ | **-19.21** | **-19.24** | **-19.32** | **-19.21** | **-19.21** | **-19.26** | **-19.21** | **-19.19** | **-19.22** |
| ${}_{0}$^e^  (95% CI) | 0.051  (0.039, 0.066) | 0.052  (0.040, 0.067) | 0.052  (0.040, 0.067) | 0.051  (0.039, 0.066) | 0.051  (0.040, 0.066) | 0.052  (0.040, 0.067) | 0.051  (0.039, 0.066) | 0.051  (0.040, 0.066) | 0.052  (0.040, 0.067) |
| $1/\delta_{TgERP}$^f^  (95% CI) | 30.12  (13.50, 103.65) | 35.49  (16.20, 129.97) | 41.11 (18.99, 163.91) | 24.56 (11.79, 65.24) | 28.74  (14.12, 78.13) | 33.06  (16.47, 92.51) | 20.74  (10.47, 47.82) | 24.17  (12.52, 56.11) | 27.69  (14.58, 65.05) |

Abbreviations: CI, confidence interval; TgERP, *Toxoplasma gondii* embryogenesis-related protein; SD, standard deviation; ELISA, enzyme-linked immunosorbent assay.

Model fitted to published data [7]. The table shows log likelihood values for each of the four force of infection models, assuming a proportion of oocyst-derived infections of 1. Parameter estimates and 95% confidence intervals are displayed for the best fitting models. Bold face signifies a statistically significant (*P* < 0.05) difference in model fit using a likelihood ratio test between the simpler and more complex model. Parameter confidence intervals were estimated by likelihood profiling [15]. Parameter set 1: sensitivity 80%, specificity 85%; set 2: sensitivity 80%, specificity 90%; set 3: sensitivity 80%, specificity 95%; set 4: sensitivity 85%, specificity 85%; set 5: sensitivity 85%, specificity 90%; set 6: sensitivity 85%, specificity 95%; set 7: sensitivity 90%, specificity 85%; set 8: sensitivity 90%, specificity 90%; set 9: sensitivity 90%, specificity 95%.

^a^Exponentially damped force of infection [1].

^b^Linear force of infection with positive intercept.

^c^Linear force of infection with zero intercept.

^d^Constant (age-independent) force of infection.

^e^Yearly per-person force of infection rate.

^f^Duration of anti-TgERP IgG in years.

**Supplementary Table S6.** **Sensitivity analysis of a range of values for sensitivity and specificity of TgERP standard deviation cut-off ELISA (schools data)**

|  | Sensitivity and specificity of TgERP SD cut-off ELISA | | | | | | | | |
| --- | --- | --- | --- | --- | --- | --- | --- | --- | --- |
|  | Set 1 | Set 2 | Set 3 | Set 4 | Set 5 | Set 6 | Set 7 | Set 8 | Set 9 |
| Model 1^a^ | -60.15 | -51.12 | -44.08 | -62.82 | -53.51 | -46.18 | -65.40 | -55.85 | -48.27 |
| Model 2^b^ | **-60.15** | **-51.12** | **-44.08** | -62.82 | **-53.51** | **-46.18** | -65.40 | **-55.85** | **-48.27** |
| Model 3^c^ | -62.14 | -53.48 | -47.41 | **-64.67*** | -55.73 | -49.34 | **-67.13*** | -57.94 | -51.28 |
| Model 4^d^ | -73.17 | -63.46 | -55.18 | -76.75 | -66.71 | -58.08 | -80.23 | -69.90 | -60.96 |
| ${}_{0}$^e^  (95% CI) | 0.011 (0.00017, 0.024) | 0.012 (0.0011, 0.025) | 0.015 (0.0033, 0.027) | - | 0.012 (0.00075, 0.024) | 0.014 (0.0029, 0.026) | - | 0.011 (0.00044, 0.024) | 0.014 (0.0025, 0.026) |
| ${}_{1}$^f^  (95% CI) | 0.0047 (0.0028, 0.0067) | 0.0045 (0.0026, 0.0066) | 0.0042 (0.0024, 0.0063) | 0.0063  (0.0052, 0.0076) | 0.0046 (0.0027, 0.0066) | 0.0043 (0.0025, 0.0063) | 0.0063  (0.0052, 0.0076) | 0.0047 (0.0028, 0.0067) | 0.0044 (0.0026, 0.0064) |
| ${1/\delta}_{TgERP}$^g^  (95% CI) | 13.88  (7.01, 40.40) | 15.95 (7.97, 50.73) | 18.90 (9.30, 69.95) | 9.70  (5.39, 21.18) | 13.09 (6.91, 33.77) | 15.44 (8.05, 43.35) | 8.25  (4.74, 16.54) | 10.98 (6.05, 24.81) | 12.91 (7.06, 30.86) |

Abbreviations: CI, confidence interval; TgERP, *Toxoplasma gondii* embryogenesis-related protein; SD, standard deviation; ELISA, enzyme-linked immunosorbent assay.

Model fitted to published data [5]. The table shows log likelihood values for each of the four force of infection models, assuming a proportion of oocyst-derived infections of 1. Parameter estimates and 95% confidence intervals are displayed for the best fitting models. Bold face signifies a statistically significant (*P* < 0.05) difference in model fit using a likelihood ratio test between the simpler and more complex model. Asterisks signify a near-significant (> 0.05 *P* < 0.10) difference in model fit between the simpler and more complex model. Parameter confidence intervals were estimated by likelihood profiling [15]. Parameter set 1: sensitivity 80%, specificity 85%; set 2: sensitivity 80%, specificity 90%; set 3: sensitivity 80%, specificity 95%; set 4: sensitivity 85%, specificity 85%; set 5: sensitivity 85%, specificity 90%; set 6: sensitivity 85%, specificity 95%; set 7: sensitivity 90%, specificity 85%; set 8: sensitivity 90%, specificity 90%; set 9: sensitivity 90%, specificity 95%.

^a^Exponentially damped force of infection [1].

^b^Linear force of infection with positive intercept.

^c^Linear force of infection with zero intercept.

^d^Constant (age-independent) force of infection.

^e^Yearly per-person force of infection rate at birth.

^f^Yearly per-person force of infection rate (this term is multiplied by age in years).

^g^Duration of anti-TgERP IgG in years.

**Supplementary Table S7.** **Sensitivity analysis of a range of values for proportion of oocyst-derived infections (population data)**

|  | Proportion of oocyst-derived infections | | | | | |
| --- | --- | --- | --- | --- | --- | --- |
|  | 0.50 | 0.60 | 0.70 | 0.80 | 0.90 | 1.0 |
| Model 1^a^ | -21.12 | -20.42 | -19.96 | -19.65 | -19.45 | -19.31 |
| Model 2^b^ | -21.30 | -20.53 | -20.02 | -19.68 | -19.46 | -19.32 |
| Model 3^c^ | -28.99 | -27.86 | -27.01 | -26.36 | -25.85 | -25.44 |
| Model 4^d^ | **-21.30** | **-20.53** | **-20.02** | **-19.68** | **-19.46** | **-19.32** |
| ${}_{0}$^e^  (95% CI) | 0.056  (0.044, 0.071) | 0.055  (0.043, 0.070) | 0.054  (0.042, 0.069) | 0.053  (0.041, 0.068) | 0.053  (0.041, 0.068) | 0.052  (0.040, 0.067) |
| $1/\delta_{TgERP}$^f^  (95% CI) | 289.95  (51.51, NA) | 121.24  (35.84, NA) | 79.35  (29.07, NA) | 59.98  (24.66, 1007.36) | 48.64  (21.45, 274.49) | 41.11  (18.99, 163.91) |

Abbreviations: CI, confidence interval; TgERP, *Toxoplasma gondii* embryogenesis-related protein; NA, not available.

Model fitted to published data [7]. The table shows log likelihood values for each of the four force of infection models. The sensitivity and specificity of the TgERP standard deviation (SD) cut-off ELISA were set to 80% and 95%, respectively. Bold face signifies a statistically significant (*P* < 0.05) difference in model fit using a likelihood ratio test between the simpler and more complex model. Parameter confidence intervals were estimated by likelihood profiling [15].

^a^Exponentially damped force of infection [1].

^b^Linear force of infection with positive intercept.

^c^Linear force of infection with zero intercept.

^d^Constant (age-independent) force of infection.

^e^Yearly per-person force of infection rate.

^f^Duration of anti-TgERP IgG in years.

**Supplementary Table S8.** **Sensitivity analysis of a range of values for proportion of oocyst-derived infections (schools data)**

|  | Proportion of oocyst-derived infections | | | | | |
| --- | --- | --- | --- | --- | --- | --- |
|  | 0.50 | 0.60 | 0.70 | 0.80 | 0.90 | 1.0 |
| Model 1^a^ | -41.18 | -40.64 | -41.38 | -42.29 | -43.21 | -44.08 |
| Model 2^b^ | **-41.18** | **-40.64** | **-41.38** | **-42.29** | **-43.21** | **-44.08** |
| Model 3^c^ | -45.84 | -45.65 | -45.92 | -46.35 | -46.86 | -47.41 |
| Model 4^d^ | -49.86 | -48.80 | -49.32 | -50.86 | -53.00 | -55.18 |
| ${}_{0}$^e^  (95% CI) | 0.021  (0.0068, 0.036) | 0.020  (0.0072, 0.035) | 0.019  (0.0062, 0.033) | 0.017  (0.0050, 0.030) | 0.016  (0.0041,  0.029) | 0.015 (0.0033, 0.027) |
| ${}_{1}$^f^  (95% CI) | 0.0043  (0.0022, 0.0063) | 0.0038 (0.0019, 0.0057) | 0.0038  (0.0018, 0.0059) | 0.0039  (0.0020, 0.0060) | 0.0041  (0.0022, 0.0062) | 0.0042 (0.0024, 0.0063) |
| $1/\delta_{TgERP}$^g^  (95% CI) | 111,420,525  (NA, NA) | 17,446,764 (NA, NA) | 132.64  (24.28, NA) | 44.74  (15.61, NA) | 26.74  (11.66, 323.23) | 18.90  (9.30, 69.95) |

Abbreviations: CI, confidence interval; TgERP, *Toxoplasma gondii* embryogenesis-related protein; NA, not available.

Model fitted to published data [5]. The table shows log likelihood values for each of the four force of infection models. The sensitivity and specificity of the TgERP standard deviation (SD) cut-off ELISA were set to 80% and 95%, respectively. Bold face signifies a statistically significant (*P* < 0.05) difference in model fit using a likelihood ratio test between the simpler and more complex model. Parameter confidence intervals were estimated by likelihood profiling [15].

^a^Exponentially damped force of infection [1].

^b^Linear force of infection with positive intercept.

^c^Linear force of infection with zero intercept.

^d^Constant (age-independent) force of infection.

^e^Yearly per-person force of infection rate at birth.

^f^Yearly per-person force of infection rate (this term is multiplied by age in years).

^g^Duration of anti-TgERP IgG in years.

**References**

1. Ades AE, Nokes DJ. Modeling age-and time-specific incidence from seroprevalence: toxoplasmosis. Am J Epidemiol **1993**; 137:1022-34.

2. Hofgärtner WT, Swanzy SR, Bacina RM, et al. Detection of immunoglobulin G (IgG) and IgM antibodies to *Toxoplasma gondii*: evaluation of four commercial immunoassay systems. J Clin Microbiol **1997**; 35:3313-15.

3. Naghili B, Abbasalizadeh S, Tabrizi S, et al. Comparison of IIF, ELISA and IgG avidity tests for the detection of anti-*Toxoplasma* antibodies in single serum sample from pregnant women. Infez Med **2017**; 25:50-6.

4. Crouch CF. Enzyme immunoassays for IgG and IgM antibodies to *Toxoplasma gondii* based on enhanced chemiluminescence. J Clin Pathol **1995**; 48:652-57.

5. Mangiavacchi BM, Vieira FP, Bahia-Oliveira LM, Hill D. Salivary IgA against sporozoite-specific embryogenesis-related protein (TgERP) in the study of horizontally transmitted toxoplasmosis via *T. gondii* oocysts in endemic settings. Epidemiol Infect **2016**; 144:2568-77.

6. Diggle PJ. Estimating prevalence using an imperfect test. Epidemiol Res Int **2011**.

7. Vieira FP, Alves Mda G, Martins LM, et al. Waterborne toxoplasmosis investigated and analysed under hydrogeological assessment: new data and perspectives for further research. Mem I Oswaldo Cruz **2015**; 110:929-35.

8. Mainar-Jaime RC, Barberan M. Evaluation of the diagnostic accuracy of the modified agglutination test (MAT) and an indirect ELISA for the detection of serum antibodies against *Toxoplasma gondii* in sheep through Bayesian approaches. Vet Parasitol **2007**; 148:122-9.

9. Casartelli-Alves L, Boechat VC, Macedo-Couto R, et al. Sensitivity and specificity of serological tests, histopathology and immunohistochemistry for detection of *Toxoplasma gondii* infection in domestic chickens. Vet Parasitol **2014**; 204:346-51.

10. Gamble HR, Dubey JP, Lambillotte DN. Comparison of a commercial ELISA with the modified agglutination test for detection of *Toxoplasma* infection in the domestic pig. Vet Parasitol **2005**; 128:177-81.

11. Garcia JL, Navarro IT, Vidotto O, et al. *Toxoplasma gondii*: comparison of a rhoptry-ELISA with IFAT and MAT for antibody detection in sera of experimentally infected pigs. Exp Parasitol **2006**; 113:100-5.

12. Shaapan RM, El-Nawawi FA, Tawfik MA. Sensitivity and specificity of various serological tests for the detection of *Toxoplasma gondii* infection in naturally infected sheep. Vet Parasitol **2008**; 153:359-62.

13. Hill DE, Chirukandoth S, Dubey JP, Lunney JK, Gamble HR. Comparison of detection methods for *Toxoplasma gondii* in naturally and experimentally infected swine. Vet Parasitol **2006**; 141:9-17.

14. Dubey JP, Thulliez P, Weigel RM, Andrews CD, Lind P, Powell EC. Sensitivity and specificity of various serologic tests for detection of *Toxoplasma gondii* infection in naturally infected sows. Am J Vet Res **1995**; 56:1030-36.

15. Bolker B, R Development Core Team. bbmle: Tools for general maximum likelihood estimation. **2010**. [https://CRAN.R-project.org/package=bbmle](https://cran.r-project.org/package=bbmle).
